# Treatment attrition and outcomes of myeloma patients in Finland: a real-world study

**DOI:** 10.2340/1651-226X.2026.46473

**Published:** 2026-10-20

**Authors:** Mervi Putkonen, Katja Närhi, Johanna Vikkula, Riikka Mattila, Marika Waltari, Jonna Eeva, Anu Partanen

**Affiliations:** a Hematology and Stem Cell Transplant Unit, Wellbeing Services County of Southwest Finland, Turku University Hospital, Turku, Finland; b Johnson & Johnson, Espoo, Finland; c Medaffcon Oy, Espoo, Finland; d Department of Medicine, Wellbeing Services County of Pirkanmaa, Tampere University Hospital, Tampere, Finland; e Department of Medicine, Wellbeing Services County of North Savo, Kuopio University Hospital, Kuopio, Finland

**Keywords:** real-world, multiple myeloma, attrition

## Abstract

**Background and purpose:**

Relapses, need for further treatment lines and significant treatment attrition are typical for multiple myeloma (MM). This retrospective study investigates attrition and outcomes among MM patients in Finland.

**Patient, material and methods:**

Study included all adult MM patients (*n* = 1733) diagnosed and treated in four hospital districts in Finland in 2013–2022. Data were collected from hospital data lakes, Statistics Finland and Social Insurance Institution. Treatment attrition was defined as death before next treatment line.

**Results:**

By the end of follow-up 31% of patients without stem cell transplantation (non-SCT) had died before initiation of second line treatment (2L), compared with 5% of stem-cell transplant (SCT) patients. The factors associating with this early attrition in the non-SCT cohort were older age (over 75 years), higher comorbidity burden, International Staging System (ISS), and (R)-ISS scores (*p* < 0.001). Furthermore, non-SCT patients were more likely administered less intensive therapy during 1L (*p* < 0.001). During the first 6 months post diagnosis in the non-SCT cohort, the most common immediate cause of death was MM. Also, during later stages MM was responsible for 54% of deaths in the non-SCT and 68% in the SCT cohorts whereas infections were linked principally with early deaths before 2L treatment.

**Interpretation:**

This study highlights significant treatment attrition across disease course and also early mortality especially in the non-SCT cohort resulting primarily from progressive MM or infections. The findings underscore the need for more tolerable and effective anti-myeloma treatments in earlier course of the disease to enhance patient survival.

## Graphical abstract

**Figure:**
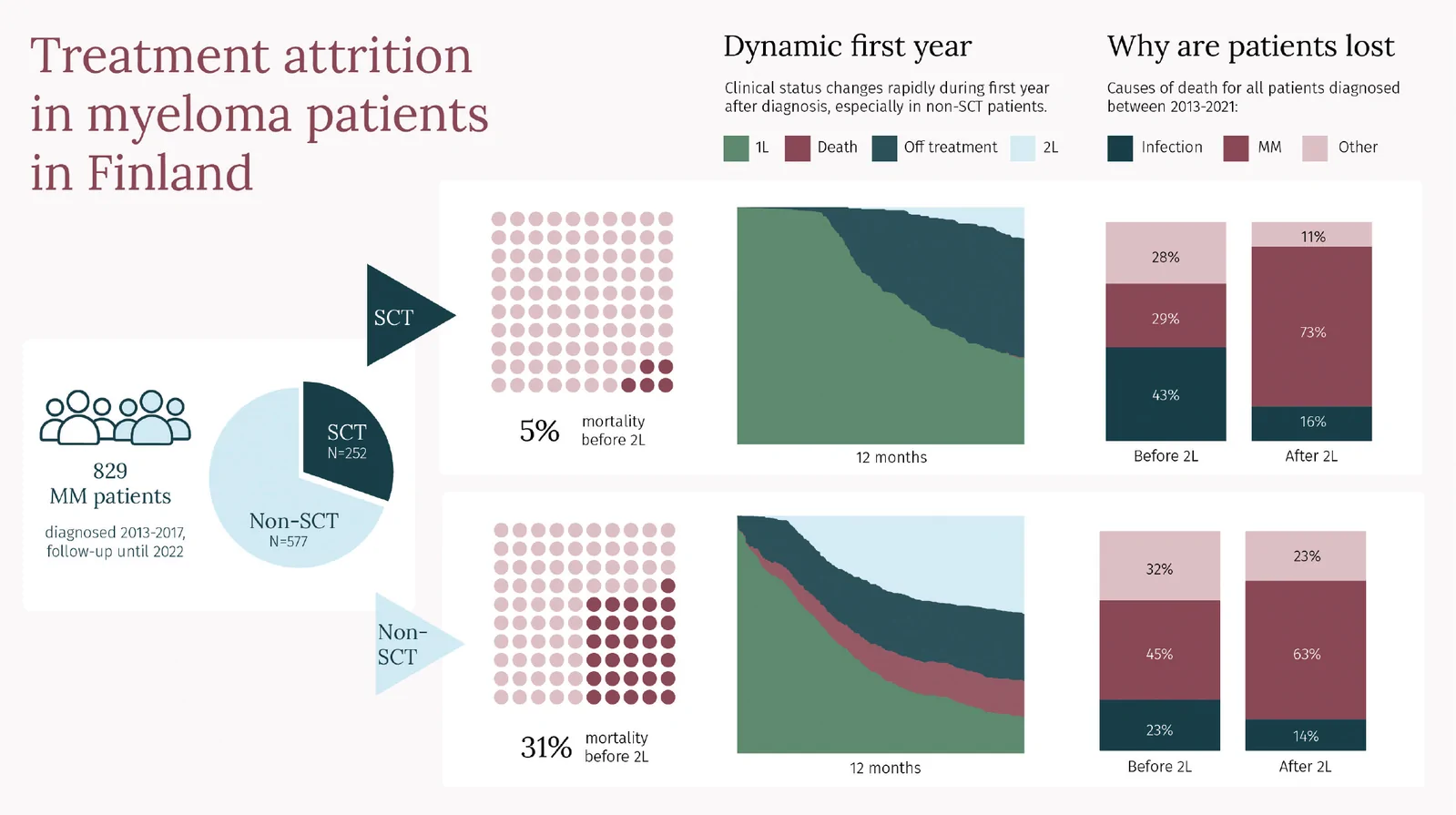

## Introduction

The treatment course of multiple myeloma (MM) typically involves series of therapies that induce remissions, followed by relapses that necessitate change of treatment used. However, a significant number of patients are unable to proceed to subsequent treatments, a phenomenon commonly referred to as treatment attrition. After each relapse the proportion of patients who receive treatment declines [1, 2].

Treatment attrition in MM has been explored in several studies from Europe [1, 3–6] and North America [7–9]. Prior publications have variously classified attrition as death before initiation of the next treatment line [8], clinical progression or deterioration without subsequent therapy [9] or composite endpoints that include death together with remaining on a line or long gaps in follow‑up [4]. Despite differing study setups and definitions for attrition, these studies consistently show that attrition rates generally increase with each successive treatment line ranging from 7 to 57% [1, 3, 4, 7–9]. Notably, attrition rates are higher among without stem cell transplantation (non-SCT) patients [3–5, 7–9]. The causes of death also vary depending on the timing of death: early deaths are more often caused by factors such as renal failure or infection, whereas disease progression is more common in later stages of the disease [10, 11]. In addition, early mortality has been associated with older age and comorbidities [12, 13].

Although overall survival has improved for stem-cell transplant (SCT)‑eligible MM patients in Finland, comparable gains have not been observed in non‑SCT patients, as shown in our recent national study [14]. This persistent gap highlights the importance of understanding how and when non‑SCT patients disengage from the treatment pathway. In Finland – as in many countries – access to novel agents and treatment sequencing is shaped by national reimbursement structures, which may influence early outcomes. Clarifying when patients are lost between treatment lines, what contributes to early deaths, and how these patterns vary across age groups is therefore essential. Accordingly, the aim of this study was to characterize the temporal dynamics of attrition and mortality in Finnish MM patients, with a particular focus on the non‑SCT population.

## Patients, material and methods

This retrospective real-world study included MM patients diagnosed (ICD-10 C90) and treated in 2013–2022 in four university hospital districts in Finland (Helsinki and Uusimaa, HUS; Pirkanmaa, PHD; Southwest Finland, HDSF; Northern Savo, HDNS [HDNS data from 2015 onwards]) covering approximately 54% of the population of Finland. For the identified cohort, specialized care data were collected from the data lakes of the respective hospital districts and complemented with data from Care register for healthcare (primary care data), Social Insurance Institution (data of reimbursed medication purchases), and Statistics Finland (data of causes of death). Patients were followed from the MM diagnosis (index date) until death or end of study (EOS), December 31, 2022, whichever came first. The cause of death data was available only through 2021 and was thus analyzed accordingly. The cohort was stratified to early and late subgroups (2013–2017 vs. 2018–2022) based on the diagnosis year and to SCT and non-SCT subgroups based on performed procedures during the follow-up. Because patients were classified as non-SCT until the date of transplantation, deaths occurring before transplantation were attributed to the non-SCT cohort. We refer to our previous publication [14] for more details of the cohort formation, collected data, and patient stratification. This study is reported in accordance with the STROBE (Strengthening the Reporting of Observational Studies in Epidemiology) guidelines [15].

### Treatment lines

For patients with SCT, the first line treatment (1L) included the induction(s), SCT, and possible consolidation and maintenance treatments since reimbursed. All other treatment lines were constructed as recommended by the current international guidelines [16] and as described in our previous study [14].

### Statistical analyses

In this study attrition was defined as death before next treatment line. However, the analyses did not account whether patient died while on or off active treatment, after progression, or due to adverse event. When analyzing the attrition rates for different treatment lines, three other treatment statuses were defined: next treatment (i.e. patient had started the next line of treatment), ongoing treatment (i.e. patient’s last treatment record was within 3 months before EOS and the patient had not started the next treatment line), and no treatment (i.e. patient had completed the treatment line and was alive at EOS).

When analyzing the attrition rates using a multistate time-to-event model, the follow-up started at the initial state (s0) (i.e. 1L treatment ongoing), from which patients were allowed to move directly to the 2nd treatment line (event) or death (event) or through a mid-state ‘no treatment’. If patient died or the 2nd treatment line started within a month from the last record of the 1L, patient moved directly from the initial state to the end state. Patients without either events were censored from the initial state (s0) or from the ‘no treatment’ at the EOS.

Immediate causes of death were analyzed similarly using a multistate model: patients started from the initial state (s0) (i.e. MM diagnosis) from which they were allowed to move to one of the mutually exclusive states of immediate cause of death: MM, other baseline malignancy, secondary malignancy, infection, cardiovascular disease (CVD), or other (See Supplemental Methods for more details). Patients alive at EOS were censored. The diagrams of both multistate models are shown in Supplementary Figure 1.

Charlson comorbidity index (CCI [17]) was defined using specialized care diagnosis data from 5 years prior to diagnosis of MM (excluding ICD-10 code C90). For other clinical variables, data at maximum ±3 months from index was used. The difference between strata was tested using chi-squared/Fisher’s exact test (categorical variables) and Kruskal-Wallis test (continuous variables). All analyses were performed with R version 4.0.3. All results with fewer than three patients were censored and reported as ‘*n* < 3’ to comply with Finnish legislation on data anonymity. Consequently, some results are presented as ranges or as ‘*n* < 5’ to prevent back-calculation of small cell counts.

## Results

### Treatment attrition and patient characteristics

A total of 1733 patients with MM were included, with a median follow-up time of 34 months (interquartile range [IQR]: 15–58 months). Treatment status and attrition were first assessed among patients diagnosed 2013–2017, across 1L–3L (Figure 1). At EOS, 70% of SCT patients had proceeded to 2L, 8% remained on 1L, 5% had died and 17% were alive without antimyeloma treatment. Attrition increased to 8% after 2L and 22% after 3L. In contrast, 31% of non-SCT patients died before 2L, 60% proceeded to 2L, 7% were alive without treatment, and only 2% had ongoing 1L treatment. Attrition remained similar after 2L but increased to 38% after 3L. Similar trends were observed among patients diagnosed in 2018–2022, although the later cohort remains immature, particularly for SCT patients, most of whom were still receiving 1L at EOS (Supplementary Figure 2).

**Figure 1:**
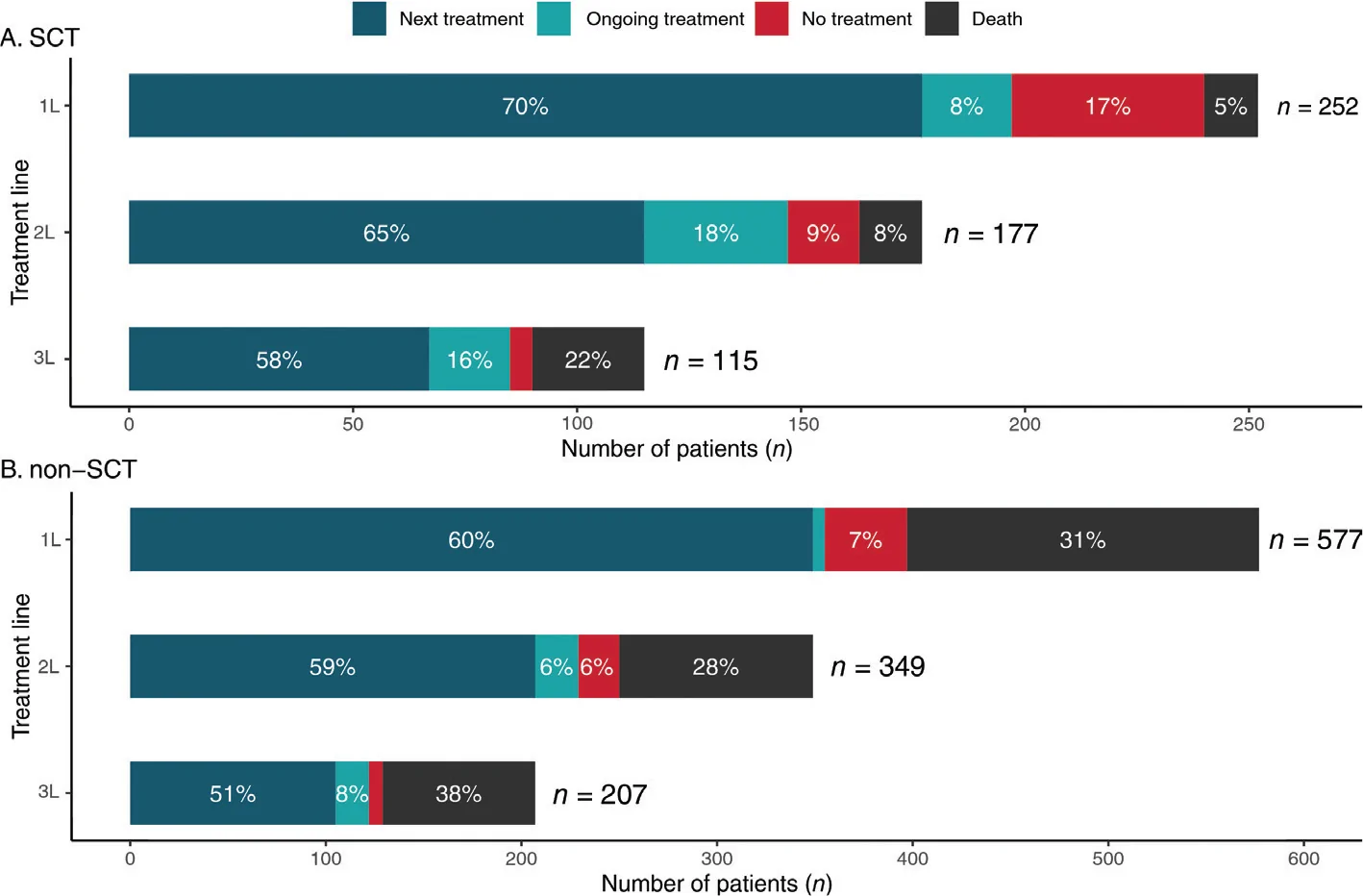
Treatment status and attrition rates for the three treatment lines (1L–3L) for SCT (upper panel) and non-SCT (lower panel) patients diagnosed between 2013 and 2017. Patients were followed until December 31, 2022. SCT: stem-cell transplant; non-SCT: without stem-cell transplantation.

Baseline characteristics by 1L treatment status were analyzed for patients diagnosed in 2013–2017 (Tables 1 and 2). Among SCT patients, no significant differences in age, CCI, International Staging System (ISS), or (R)-ISS were observed between those who proceeded to 2L and those who died before 2L, although the low attrition rate limited statistical power. In contrast, non-SCT patients who died before 2L were older and had higher CCI, ISS, and R-ISS than those who proceeded to 2L (*p* < 0.001). Similar patterns were seen in non-SCT patients diagnosed in 2018–2022, among whom death before 2L was associated with older age (*p* < 0.001) and higher ISS (*p* = 0.04; Supplementary Tables 1–2).

**Table 1:** Baseline characteristics of SCT MM patients diagnosed between 2013 and 2017 (N = 252) based on their status after 1st SCT treatment.

Variable		Subsequent treatment	No treatment	Ongoing treatment	Death	Missing %
***n***		177	43	20	12	
**Age at index, years, median [IQR]**		61 [56, 67]	58 [52, 63]	61 [56, 67]	59 [53, 64]	
**Age at index, years, *N* (%)**	**< 65**	113 (64)	35 (81)	13 (65)	10–11	0
	**≥ 65**	64 (36)	8 (19)	7 (35)	< 3	
**Sex, *N* (%)**	**Female**	85 (48)	27 (63)	11 (55)	5 (42)	0
**CCI, *N* (%)**	**0**	135 (76)	30 (70)	14 (70)	11 (92)	0
	**1–2**	28 (16)	11–12	≤ 5	< 3	
	**3+**	14 (8)	< 3	≤ 3	< 3	
**ECOG, *N* (%)**	**0**	23 (30)	6 (40)	3 (50)	< 3	60
	**1**	35 (46)	6 (40)	3 (50)	< 3	
	**2**	14 (18)	< 3	0 (0)	0 (0)	
	**3–4**	5 (7)	< 3	0 (0)	0 (0)	
High risk cytogenetics*, *N* (%)		33 (27)	3 (11)	< 3	<3	32
**ISS, *N* (%)**	**I**	47 (29)	12 (31)	4 (25)	3 (27)	10
	**II**	68 (42)	13 (33)	9 (56)	5 (46)	
	**III**	46 (29)	14 (36)	3 (19)	3 (27)	
**R-ISS, *N* (%)**	**I**	20 (14)	4 (13)	< 3	< 3	23
	**II**	99 (70)	22 (71)	11 (85)	7 (78)	
	**III**	22 (16)	5 (16)	< 3	< 3	
**Length of follow-up, months, median [IQR]**		76 [54, 93]	81 [75, 101]	76 [70, 92]	23 [18, 30]	

‘Subsequent treatment’ indicates that the patient has started the next line of treatment. ‘Ongoing treatment’ indicates that the treatment is ongoing as of EOS (December 31, 2022). ‘No treatment’ indicates the patient has completed the treatment line, but has not started a new treatment, and not died by EOS (December 31, 2022). ‘Death’ indicates that the patient has died before starting the next treatment. SCT: stem-cell transplant; MM: multiple myeloma; IQR; interquartile range; CCI: Charlson comorbidity index; EOS: end of study; ECOG: Eastern Cooperative Oncology Group performance status.

* High risk cytogenetic changes include *t*(4;14), *t*(14;16), and del(17p).

**Table 2:** Baseline characteristics of non-SCT MM patients diagnosed between 2013 and 2017 (N = 577) based on their status after 1st treatment.

Variable		Subsequent treatment	No treatment	Ongoing treatment	Death	Missing %
***N***		349	42	6	180	
**Age at index, years, median [IQR]**		74 [70, 78]	71 [61, 76]	68 [56, 75]	77 [71, 81]	0
**Age at index, years, *N* (%)**	**< 65**	43 (12)	12 (29)	< 3	21 (12)	0
	**65–75**	182 (52)	20 (48)	< 3	64 (36)	
	**> 75**	124 (36)	10 (24)	< 3	95 (53)	
**Sex, *N* (%)**	**Female**	167 (48)	19 (45)	3 (50)	90 (50)	0
**CCI, *N* (%)**	**0**	238 (68)	16 (38)	< 5	88 (49)	0
	**1–2**	83 (24)	20 (48)	< 3	61 (34)	
	**3+**	28 (8)	6 (14)	< 3	31 (17)	
**ECOG, *N* (%)**	**0**	17 (14)	4 (25)	< 3	5 (14)	69
	**1**	52 (41)	6 (38)	0 (0)	12 (32)	
	**2**	39 (31)	< 5	0 (0)	9 (24)	
	**3–4**	18 (14)	< 3	0 (0)	11 (30)	
High risk cytogenetics*, *N* (%)		45 (25)	4 (19)	< 3	7 (14)	55
**ISS, *N* (%)**	**I**	31 (12)	< 5	< 3	7 (7)	32
	**II**	130 (48)	15 (75)	0 (0)	33 (33)	
	**III**	108 (40)	< 3	< 3	61 (60)	
**R-ISS, *N* (%)**	**I**	9 (4)	< 3	< 3	4 (6)	44
	**II**	175 (77)	16 (84)	< 3	42 (58)	
	**III**	44 (19)	< 3	< 3	26 (36)	
**Length of follow-up, months, median [IQR]**		49 [28, 74]	92 [73, 112]	97 [77, 103]	15 [3, 38]	0

‘Death’ indicates that the patient has died before starting the next treatment. ‘Subsequent treatment’ indicates that the patient has started the next line of treatment. ‘Ongoing treatment’ indicates that the treatment is ongoing as of EOS (December 31, 2022). ‘No treatment’ indicates the patient has completed the treatment line, but has not started a new treatment, and not died by EOS (December 31, 2022). non-SCT: without stem cell transplantation; MM: multiple myeloma; IQR: interquartile range; CCI: Charlson comorbidity index; EOS: end of study; ECOG: Eastern Cooperative Oncology Group performance status.

* High risk cytogenetic changes include *t*(4;14), *t*(14;16), and del(17p).

Patients who remained alive without active antimyeloma treatment after 1L had a median follow-up time of 81 months in the SCT cohort (IQR: 75, 101) and 92 months in the non-SCT cohort (IQR: 73, 112). In the SCT group, patients were younger, (81% <65 years), compared with those who initiated 2L (64% <65 years). Similarly, in the non-SCT group, patients alive without further treatment were younger (29% <65 years) than those who initiated 2L or those who died (both 12% <65 years) and had lower CCI and R-ISS scores. The underlying clinical reason for the absence of additional therapy cannot be determined from the data, and likely reflects heterogeneous patient trajectories.

Age-stratified analyses showed low attrition before 2L across SCT age groups, whereas attrition was consistently higher in the non-SCT cohort and highest among patients > 75 years, 41% of whom died before 2L (Figure 2). Among non-SCT patients, those < 65 years were more often without active pharmacological treatment than those aged 65–75 years (under 20% vs. under 10%; *p* < 0.001) and less often initiated 2L before EOS (55 vs 68%), although mortality was similar between these two age groups.

**Figure 2:**
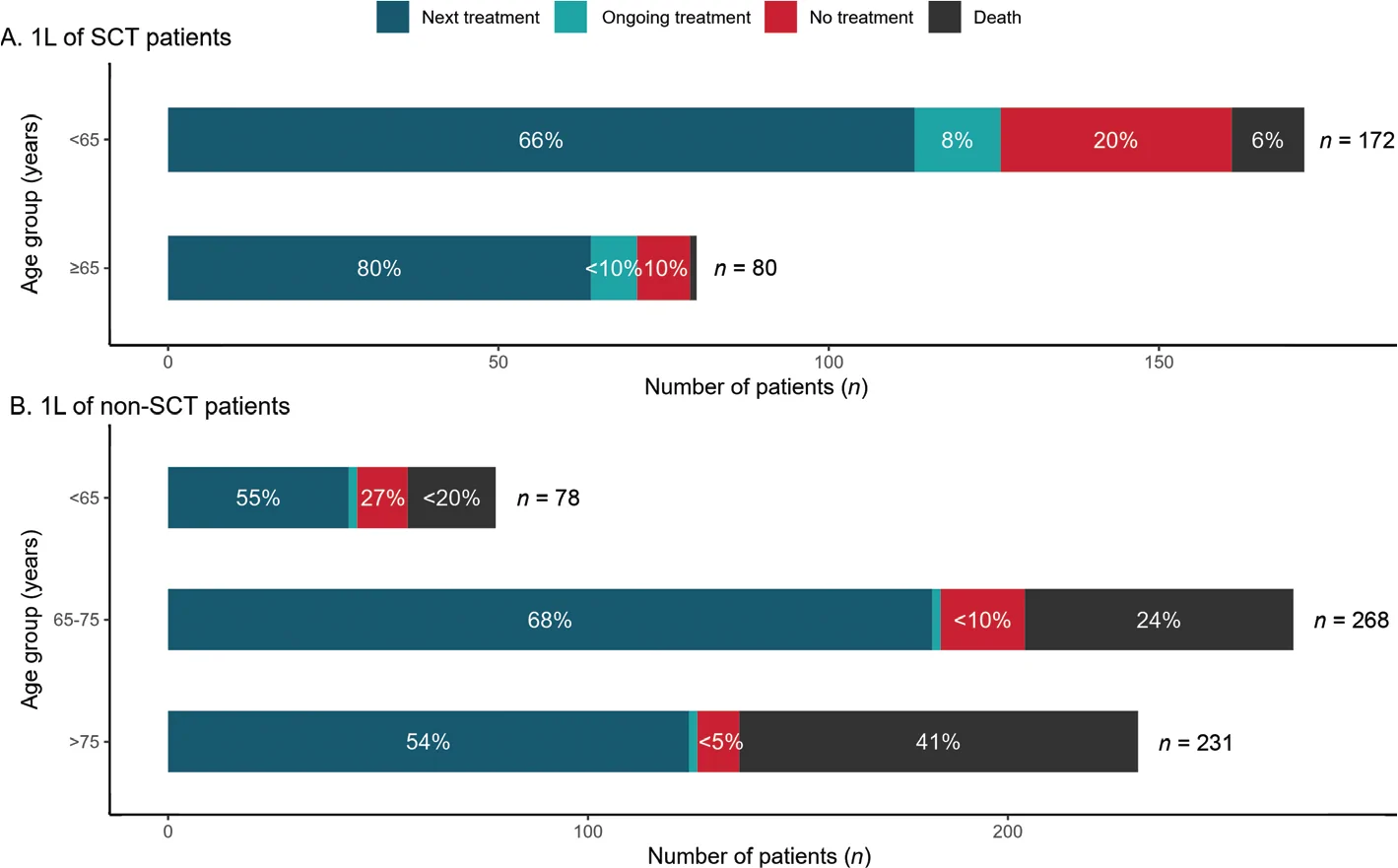
Treatment status and attrition rates after 1L by age group for the SCT (upper panel) and the non-SCT (lower panel) patients diagnosed between 2013 and 2017. SCT: stem-cell transplant; non-SCT: without stem-cell transplantation.

Baseline characteristics stratified by age indicated that non-SCT patients <65 years had higher comorbidity burden than SCT patients of the same age (CCI > 0 in 48 vs. 31%, *p* = 0.001), whereas younger non-SCT patients tended to have lower ISS and R-ISS stages than older non-SCT patients (Supplementary Table 3). Overall, early attrition before 2L was most consistently associated with older age, non-transplant status, higher CCI, and/or poorer prognosis based on ISS/R-ISS.

### First-line treatment intensity

Treatment intensity was next analyzed in relation to 1L attrition (Table 3). For a comprehensive overview of how these patients were treated, please see our previous publication [14]. Of non-SCT patients who died before 2L, 72% were treated with doublet therapy, defined as one myeloma-specific medication combined with a steroid, compared with 57% of those who proceeded to subsequent treatment (*p* = 0.001). In contrast, induction intensity did not differ significantly by treatment status among SCT patients, 74% of whom received triplet or quadruplet therapy (*p* = 0.77).

**Table 3:** 1L treatment by 1L treatment status for the non-SCT and SCT-patient cohorts diagnosed between 2013 and 2017.

Treatment		Non-SCT, *N* = 577				SCT, *N* = 252			
		Next treatment	No treatment	Ongoing treatment	Death	Next treatment	No treatment	Ongoing treatment	Death
*N*		349	42	6	180	177	43	20	12
**1L treatment combination**	B ± d/p	4 (1)	5 (12)	0 (0)	10 (6)	0 (0)	0 (0)	0 (0)	0 (0)
	C ± d/p	5 (1)	8 (19)	0 (0)	26 (14)	4 (2)	< 3	< 3	0 (0)
	CAvd/p	< 3	3 (7)	< 3	7 (4)	0 (0)	0 (0)	0 (0)	0 (0)
	M ± d/p	25 (7)	< 3	0 (0)	22 (12)	0 (0)	0 (0)	0 (0)	0 (0)
	R ± d/p	21 (6)	6 (14)	< 3	14 (8)	3 (2)	0 (0)	0 (0)	0 (0)
	T ± d/p	6 (2)	0 (0)	0 (0)	4 (2)	0 (0)	0 (0)	0 (0)	0 (0)
	TM ± d/p	5 (1)	0 (0)	0 (0)	< 3	0 (0)	0 (0)	0 (0)	0 (0)
	V ± d/p	136 (39)	9 (21)	0 (0)	50 (28)	40 (23)	12 (28)	3 (15)	< 3
	VC ± d/p	56 (16)	4 (10)	< 3	9 (5)	102 (58)	22 (51)	14 (70)	7 (58)
	VM ± d/p	70 (20)	< 3	0 (0)	15 (8)	0 (0)	0 (0)	0 (0)	0 (0)
	VR ± d/p	10 (3)	< 3	< 3	8 (4)	0 (0)	0 (0)	< 3	< 3
	VTd	5 (1)	0 (0)	0 (0)	0 (0)	5 (3)	4 (9)	0 (0)	< 3
**Type of 1L therapy**	doublet	198 (57)	30 (71)	< 5	129 (72)	47 (27)	13 (30)	4 (20)	< 3
	triplet/ quadruplet	151 (43)	12 (29)	< 5	51 (28)	130 (73)	30 (70)	16 (80)	10–11

Treatment combinations with < 5 patients in all groups were omitted. For SCT patients, only the first induction treatment medicines are reported. No quadruplet regimen was administered to 5 or more patients in any treatment-status group. SCT: stem-cell transplant; non-SCT: without stem-cell transplantation.

d: dexamethasone; p: predniso(lo)ne; B: bendamustine; C: cyclophosphamide; A: doxorubicin; V: bortezomib; M: melphalan; R: lenalidomide; T: thalidomide; v: vincristine.

Across the full study period, doublet therapy was most frequent among non-SCT patients who died before 2L in all age groups (Supplementary Table 4). Among patients <75 years, doublets were more common among those who died before 2L or remained without further treatment, whereas triplets were more common among those with ongoing or subsequent treatment. In contrast, among patients ≥75 years, doublets predominated across treatment statuses. Treatment patterns differed significantly across age groups (*p* < 0.05).

### Temporal dynamics of first-line outcomes

Temporal dynamics of 1L outcomes were examined using a multistate model (Figure 3, Supplementary Figure 1; Methods). Transitions beyond 2L, including death after 2L and initiation of 3L, were not included.

**Figure 3:**
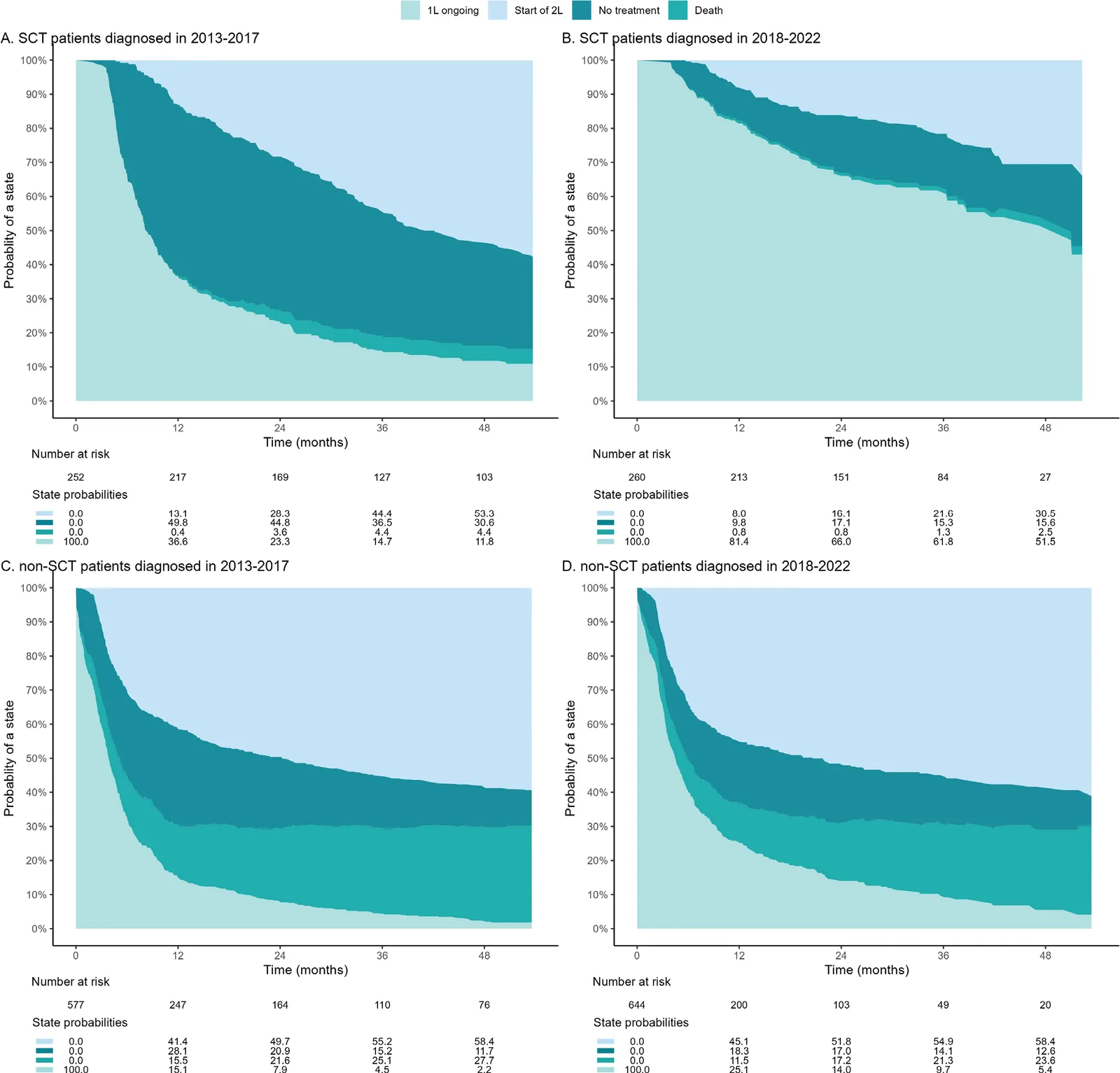
A multistate model for attrition and treatment statuses in the SCT (upper panels) and the non-SCT patient groups (lower panels) after starting 1L treatment for patients diagnosed between 2013 and 2017 (left panels) and between 2018 and 2022 (right panels). SCT: stem-cell transplant; non-SCT: without stem-cell transplantation.

Among SCT patients diagnosed in 2013–2017, attrition emerged mainly after 12 months, whereas in the non-SCT cohort deaths occurred within the first months and increased markedly after 6 months (Figure 3). Median time to 2L initiation or death was 37 months (95% CI: 33–45) in SCT patients and 8 months (95% CI: 7–11) in non-SCT patients. By 48 months, only a minority remained on 1L. Patterns were similar in non-SCT patients diagnosed in 2018–2022, whereas SCT patients in the later cohort had a more prolonged first-line trajectory.

Age-stratified multistate models showed broadly similar trajectories within SCT and non-SCT cohorts (Supplementary Figures 4–6). However, non-SCT patients <65 years more often remained without active antimyeloma treatment at 12 months (37%) than those aged 65–75 or >75 years. Despite younger, non‑SCT patients under 65 years had an overall survival similar to that of non-SCT patients aged 65–75 years (54.2 months [95% CI: 35.9–91.5] vs. 56.2 months [95% CI: 48.0–63.0]) and longer than that of patients >75 years (36.3 months [95% CI: 33.3–39.4]).

### Causes and timing of death

Across follow-up, MM was the most common immediate cause of death (Figure 4). Among patients diagnosed in 2013–2017, MM accounted for 54% of deaths in the non-SCT cohort and 68% in the SCT cohort, followed by infections (17 and 18%) and CVD (10 and 8%), respectively. Secondary malignancies resulted in death for 5 and <5%, and other baseline malignancy accounted for 5% of deaths in the non-SCT cohort and 0% in the SCT cohort. Renal failure contributed a small proportion of deaths in the non-SCT cohort with acute and chronic failure together accounting for 2%. No renal-failure deaths were recorded in the SCT-cohort. Among infection-related deaths in the non-SCT cohort, 50% were due to pneumonia and 26% to septicemia. For cardiovascular deaths in non‑SCT patients, the most frequent causes were chronic ischemic heart disease (25%), acute myocardial infarction (25%), and intracerebral hemorrhage (13%).

**Figure 4:**
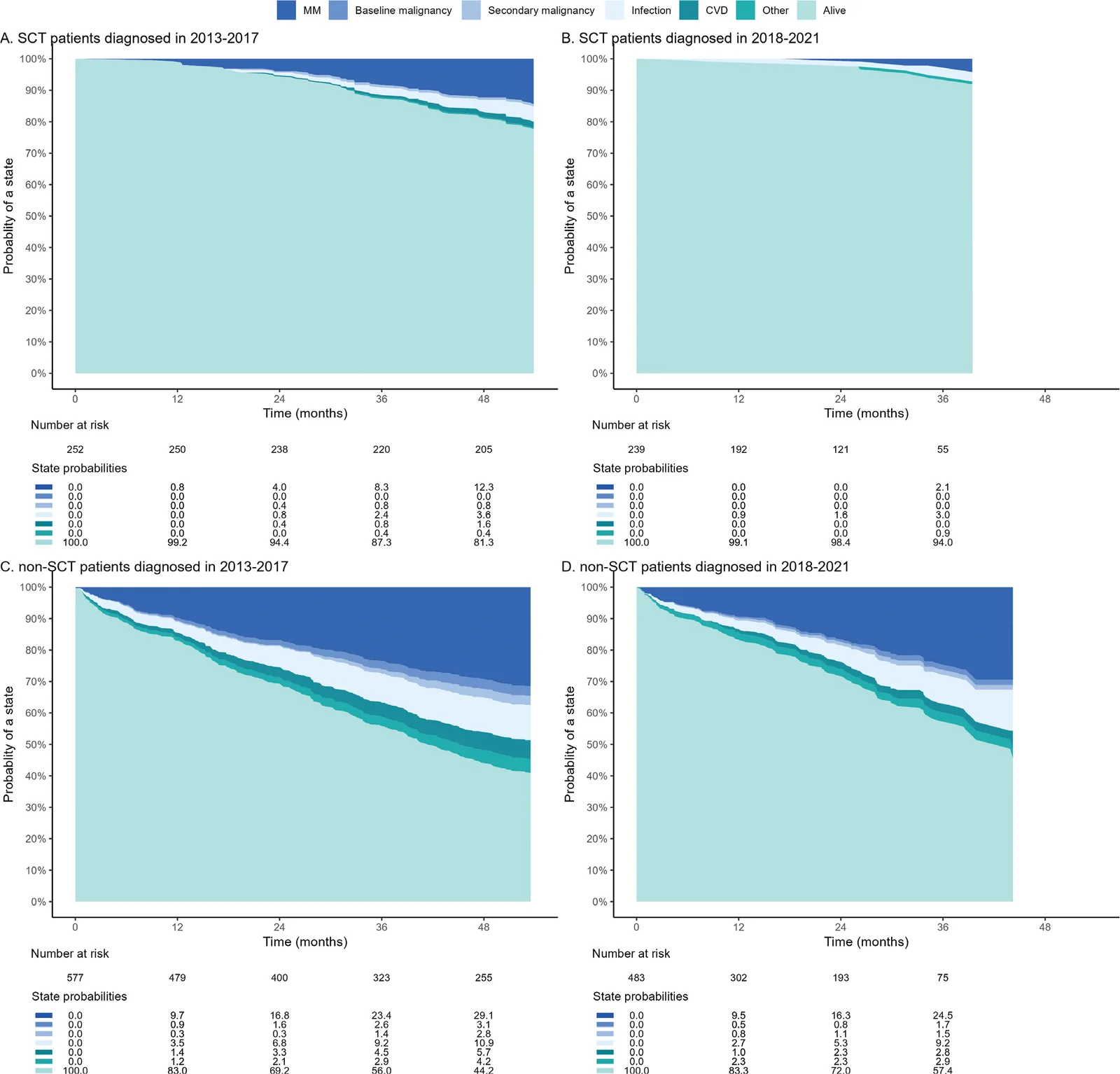
Mortality and the immediate causes of death for SCT (upper panels) and non-SCT (lower panel) patients for 2013–2017 cohorts (left panels) and 2018–2021 cohorts (right panels). SCT: stem-cell transplant; non-SCT: without stem-cell transplantation; MM: multiple myeloma; CVD: cardiovascular disease.

Cause-of-death distributions did not differ significantly by age in the SCT cohort (Supplementary Figure 7). In the non-SCT cohort, patients <65 years had a higher proportion of deaths due to other baseline malignancy (14%) compared to those aged 65–75 years (5%) and those over 75 years (3%). MM was a less frequent cause of death among <65 years (46%) compared with those aged 65–75 years (55%), and 75 years or older (57%) (*p* = 0.018 across immediate causes; Supplementary Figure 8). No major changes in cause-of-death distribution were observed between diagnosis periods for either the SCT or the non-SCT cohorts.

Cause-of-death distribution differed by timing of death in both non-SCT (*p* < 0.001) and SCT (*p* = 0.001) cohorts (Supplementary Table 5). Infection-related deaths were more frequent before 2L than after 2L initiation, decreasing from 23 to 14% in non-SCT patients and from 43 to 16% in SCT patients. Conversely, deaths due to MM increased after 2L initiation, from 45 to 63% in non-SCT patients and from 29 to 73% in SCT patients.

Within 6 months of diagnosis, 23% of deaths in the non-SCT cohort were infection related, compared with 17% after 6 months (Supplementary Table 6).

## Discussion and conclusion

This large retrospective real-world study shows that treatment attrition is substantial and under-recognized among Finnish MM patients, particularly in the non-SCT cohort. Attrition rates also increased with each subsequent line of therapy, underscoring the vulnerability and poor prognosis of patients with high-risk disease and comorbidity burden across all age groups. In the non‑SCT cohort, older age, higher comorbidity burden, and more advanced disease stage were associated with death before 2L. In contrast, early attrition in the SCT cohort was low, although attrition also increased with successive lines of therapy. MM remained the leading cause of death for both SCT and non-SCT patients at all time points. Infections were second most common cause overall, but their relative contribution was greater among deaths before 2L.

Our findings are broadly consistent with earlier real-world studies showing increasing attrition across successive treatment lines and particularly high attrition among older and non-SCT patients [4–6, 8, 9]. Although definitions of attrition differs between these studies, the magnitude and trajectory of attrition observed in Finland align well with the patterns reported across Western countries. Furthermore, recent national real-world evidence from Europe [10] reported mortality patterns closely aligned with ours.

In our earlier analysis, survival improved over time among SCT-eligible patients, but not among non-SCT patients [14]. The persistently high attrition observed in this study among non‑SCT patients – and the fact that these levels show little change between patients diagnosed in 2013–2017 and 2018–2022 – likely contributes to the lack of OS improvement previously reported. Other recent real‑world cohorts have also shown that attrition patterns are strongly influenced by patient characteristics and treatment era, although trends differ across studies [4, 5, 8].

The multistate analysis highlights the vulnerability of non-SCT patients early after diagnosis, with rapid transitions between treatment states and early deaths across age groups. In contrast, the SCT cohort showed a more stable early course, particularly among patients diagnosed in 2018–2022, possibly reflecting increased use of the maintenance therapy. The relatively large off‑treatment proportion in the non‑SCT group is consistent with the use of fixed‑duration first‑line regimens, especially in earlier calendar years. Together, this early instability highlights the importance of selecting tolerable, patient‑appropriate first‑line strategies and providing proactive supportive care to stabilize the early treatment course. Importantly, both our study and previously published real‑world studies reflect an era before the broad adoption of anti‑CD38–based frontline combinations, which have shown reduced attrition with manageable safety also in frail non-SCT populations [18, 19]. Whether broader use of contemporary frontline combinations [20] will translate into lower real‑world attrition in Finland remains to be determined as treatment access continues to evolve.

Direct evidence tying specific regimens to attrition is limited, as randomized trials typically report PFS/OS and rarely quantify deaths before the next line. During the study period, Finnish treatment practices largely followed the prevailing EHA–EMN recommendations [21], which emphasized regimens based on lenalidomide, bortezomib, or alkylating agents, given the treatment access and reimbursement conditions of the time. In our data, doublets were more common among patients who died before 2L. This pattern may reflect treatment recommendations, preferred regimens and patient-specific treatment selection during the study period, rather than causal effect. Nevertheless, registry data from Austria showed lower‑intensity regimens were associated with higher attrition [4], while long‑horizon data from Spain indicate that attrition has declined across treatment lines over successive eras [5].

In this study, patients who died before reaching 2L were more likely to succumb to infections compared to those who died after starting 2L. Recent studies in Finland [11] and Sweden [22] also highlighted an increased risk of dying from an infection early in the disease course. Taken together, the broad range of causes of death already apparent in the first months suggests that strategies to reduce early mortality need to extend beyond effective antimyeloma therapy to include careful frailty assessment, management of treatment‑related toxicities, and robust infection prevention measures.

Several limitations should be considered for this retrospective analysis. Firstly, as in other attrition studies [4, 8, 9], we included only patients who received at least one treatment line and therefore could not capture attrition before treatment initiation. Secondly, although attrition was defined as death before initiation of the next treatment line, the study could not distinguish the clinical circumstances leading to attrition, such as disease progression or other factors. Classification into the SCT cohort only after transplantation introduces immortal time bias. The cytogenetic data were incomplete and varied between hospital districts limiting assessment of high-risk genetics and R-ISS. Finally, registry-based analyses depend on the completeness and accuracy of routinely recorded data. Strengths of the study include large national coverage of data covering 54% of the Finnish population, availability of good quality data from EHRs from hospital data lakes that was enriched by linking data from national registries to include reimbursed drug purchases and causes of death, ensuring a robust and comprehensive data collection.

In conclusion, this nationwide real‑world analysis of MM in Finland demonstrates that treatment attrition is substantial, particularly among patients in the non‑SCT group, and already begins during frontline therapy. Older age, higher comorbidity burden and advanced disease stage were consistently associated with death before 2L, and early treatment trajectories were markedly dynamic in non‑SCT patients, with rapid transitions into off‑treatment, early movement to 2L, and early deaths across age groups. By examining attrition together with causes of death over time, our study provides a more granular view of when patients are lost from the treatment pathway and shows that both disease progression and infections contribute meaningfully for this at all time points. These findings underscore the need for careful frailty assessment, supportive care, and individualized first‑line strategies to improve early outcomes, especially for the non‑SCT population.

## Supplementary Material







## Data Availability

This study is based on secondary use of healthcare register data. Hence, data cannot be shared openly.
