# Supplementary material for: Treatment attrition and outcomes of myeloma patients in Finland: a real-world study
Source: Acta Oncol. 2026 Oct 20;65:46473. doi: 10.2340/1651-226X.2026.46473 (PMC13688295; doi:10.2340/1651-226X.2026.46473)
Supplement: Supplementary file 1 [file AO-65-46473-s1.pdf]

Supplementary material has been published as submitted. It has not been copyedited, or typeset by Acta Oncologica

Supplemental Methods

Immediate causes of death were grouped based in ICD-10 codes as follows:

- Multiple myeloma (MM): C90
- Other baseline malignancy: ICD-10 codes starting with C and the diagnosis of the malignancy recorded as cause of death happened before the diagnosis of MM
- Secondary malignancy: ICD-10 codes starting with C and the diagnosis of the malignancy recorded as cause of death happened after the diagnosis of MM
- Infections: ICD-10 codes starting with A, B, and J, as well as N10, N30, and U07.1
- Cardiovascular diseases (CVD): ICD-10 codes starting with I
- Other: all the rest of the ICD-10 codes not mentioned above

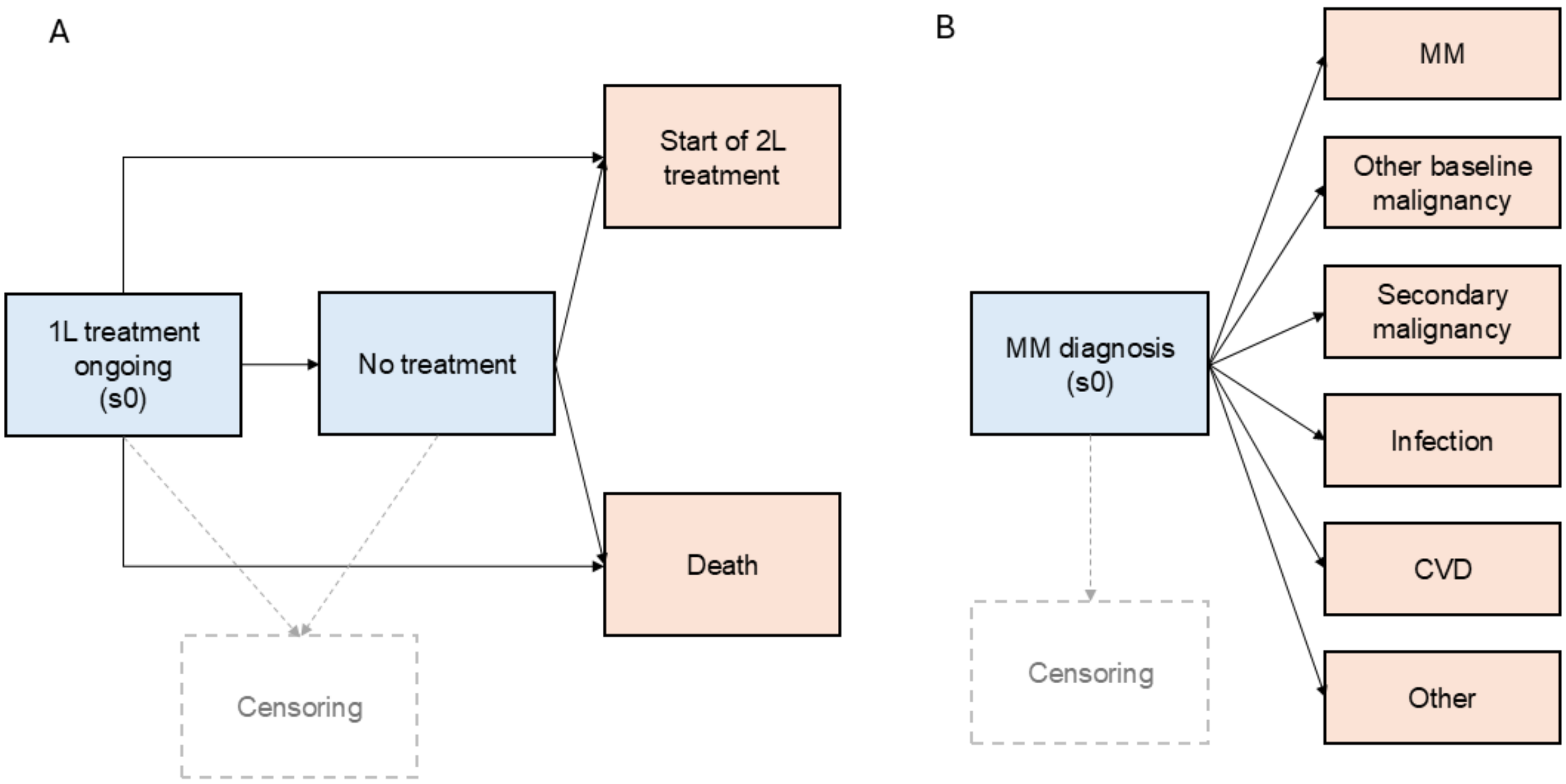

Supplementary Figure 1. *Multistate model diagram for the states after the first treatment line (A) and for the immediate causes of death (B). Patients start at the initial state (s0) and can move only to the direction of the arrows. The orange boxes indicate the final states (events), and the blue boxes indicate states from where patients were censored, if they did not get any of the end state events. MM=multiple myeloma; CVD=cardiovascular disease; 1L=first treatment line; 2L=second treatment line.*

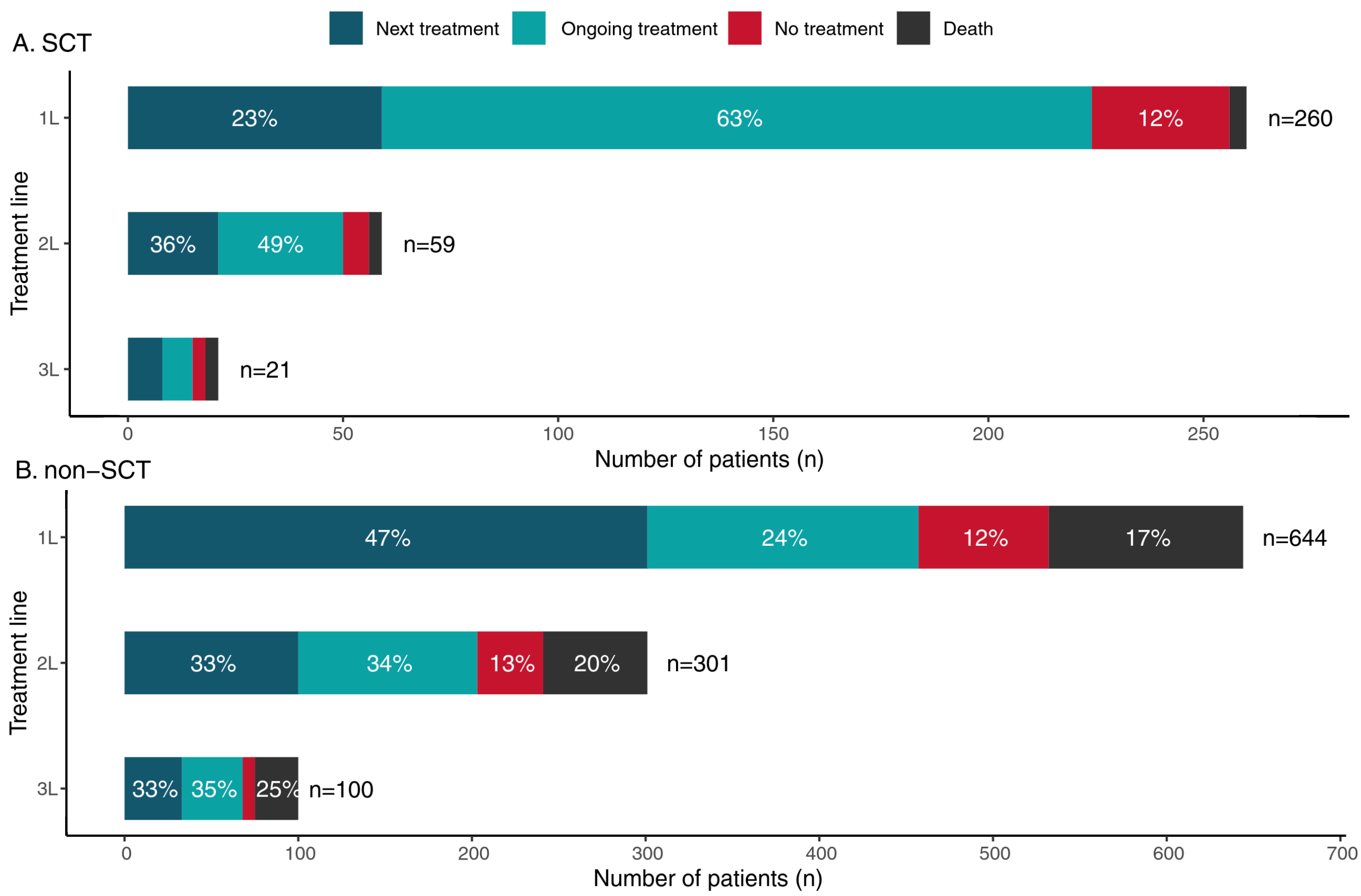

Supplementary Figure 2. *Treatment status and attrition rates by treatment for SCT (upper panel) and non-SCT (lower panel) patients diagnosed between 2018-2022 for the first three treatment lines (1L-3L). "Subsequent treatment" indicates that the patient has started the next line of treatment. "Ongoing treatment" indicates that the treatment is ongoing as of EOS (December 31, 2022). "No treatment" indicates the patient has completed the treatment line, but has not started a new treatment, and not died by EOS (December 31, 2022). "Death" indicates that the patient has died before starting the next treatment.*

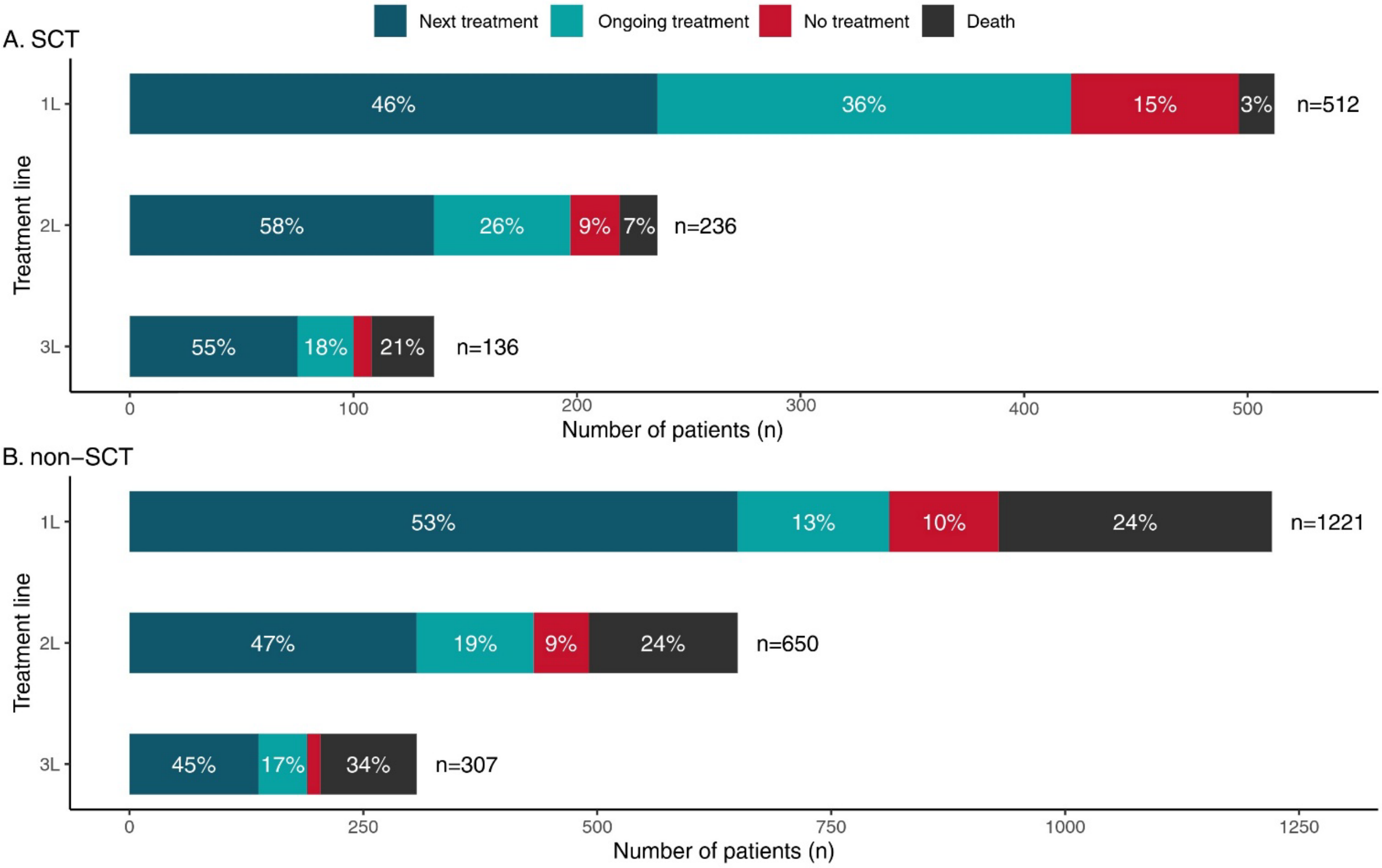

Supplementary Figure 3. *Treatment status and attrition rates by treatment for SCT (upper panel) and non-SCT (lower panel) patients diagnosed between 2013-2022 by age group. "Subsequent treatment" indicates that the patient has started the next line of treatment. "Ongoing treatment" indicates that the treatment is ongoing as of EOS (December 31, 2022). "No treatment" indicates the patient has completed the treatment line, but has not started a new treatment, and not died by EOS (December 31, 2022). "Death" indicates that the patient has died before starting the next treatment.*

Supplementary Table 1. *Baseline characteristics of non-SCT MM patients diagnosed between 2018-2022 based on their status after 1L. "Subsequent treatment" indicates that the patient has started the next line of treatment. "Ongoing treatment" indicates that the treatment is ongoing as of EOS (December 31, 2022). "No treatment" indicates the patient has completed the treatment line, but has not started a new treatment, and not died by EOS (December 31, 2022). "Death" indicates that the patient has died before starting the next treatment.*

**Variable**		**Subsequent treatment**	**No treatment**	**Ongoing treatment**	**Death**	**Missing %**
**n**		301	75	156	112	
**Age at index, years, median [IQR]**		74 [68, 79]	76 [63, 82]	76 [71, 80]	79 [72, 85]	0
**Age at index, years, N (%)**	**<65**	45 (15)	20 (27)	15 (10)	12 (11)	0
	**65-75**	142 (47)	16 (21)	65 (42)	29 (26)	
	**>75**	114 (38)	39 (52)	76 (49)	71 (63)	
**Sex, N (%)**	**female**	142 (47)	43 (57)	75 (48)	54 (48)	0
**CCI, N (%)**	**0**	185 (62)	48 (64)	103 (66)	57 (51)	0
	**1-2**	80 (27)	19 (25)	38 (24)	36 (32)	
	**3+**	36 (12)	8 (11)	15 (10)	19 (17)	
**ECOG, N (%)**	**0**	23 (16)	6 (17)	16 (19)	0 (0)	53
	**1**	48 (33)	12 (34)	29 (34)	6 (16)	
	**2**	44 (31)	9 (26)	25 (29)	15 (41)	
	**3-4**	29 (20)	8 (23)	15 (18)	16 (43)	
**High risk cytogenetics*, N (%)**		40 (24)	3 (10)	20 (18)	9 (20)	45
**ISS, N (%)**	**I**	36 (15)	7 (14)	18 (15)	<3	23
	**II**	98 (40)	26 (53)	53 (43)	34-35	
	**III**	113 (46)	16 (33)	51 (42)	43 (54)	
**RISS, N (%)**	**I**	17 (8)	<3	9 (8)	<3	34
	**II**	142 (69)	31 (76)	84 (75)	47 (69)	
	**III**	46 (22)	8-9	19 (17)	19-20	
**Length of follow-up, months, median [IQR]**		24 [13, 40]	28 [17, 40]	8 [4, 25]	7 [2, 22]	

**High risk cytogenetic changes include t(4;14), t(14;16), and del(17p).*

Supplementary Table 2. *Baseline characteristics of SCT MM patients diagnosed between 2018-2022 based on their status after 1L. "Subsequent treatment" indicates that the patient has started the next line of treatment. "Ongoing treatment" indicates that the treatment is ongoing as of EOS (December 31, 2022). "No treatment" indicates the patient has completed the treatment line, but has not started a new treatment, and not died by EOS (December 31, 2022). "Death" indicates that the patient has died before starting the next treatment. "death" was not analyzed for SCT patients due to small number of patients.*

**High risk cytogenetic changes include t(4;14), t(14;16), and del(17p).*

		**Subsequent treatment**	**No treatment**	**Ongoing treatment**
N		59	32	165
**Age at index, years, median [IQR]**		62 [53, 67]	63 [59, 69]	62 [56, 68]
**Age at index, years, N (%)**	**<65**	37 (63)	19 (59)	106 (64)
	**≥65**	22 (37)	13 (41)	59 (36)
**Sex, N (%)**	**female**	28 (48)	12 (38)	72 (44)
**CCI, N (%)**	**0**	49 (83)	27 (84)	125 (76)
	**1-2**	3 (5)	<5	27 (16)
	**3+**	7 (12)	<3	13 (8)
**ECOG, N (%)**	**0**	6 (18)	4 (27)	41 (42)
	**1**	11 (32)	6 (40)	37 (38)
	**2**	12 (35)	<5	14 (14)
	**3-4**	5 (15)	<3	5 (5)
**High risk cytogenetics*, N (%)**		15 (32)	<3	37 (29)
**ISS, N (%)**	**I**	9 (16)	8 (30)	34 (23)
	**II**	27 (48)	14 (52)	74 (49)
	**III**	20 (36)	5 (19)	43 (29)
**RISS, N (%)**	**I**	6 (11)	4 (19)	18 (13)
	**II**	33 (62)	17 (81)	99 (72)
	**III**	14 (26)	0 (0)	21 (15)
**Length of follow-up, months, median [IQR]**		39 [28, 52]	41 [35, 54]	29 [20, 42]

Supplementary Table 3. *Baseline characteristics of MM patients diagnosed between 2013-2022 based on their status after 1L. See separate Excel file.*

Supplementary Table 4. *First-line treatment intensity (doublet vs triplet/quadruplet) stratified by age group, SCT vs non-SCT cohorts and treatment status after 1L. See separate Excel file.*

Supplementary Table 5. *Causes of death by timing of death for all patients (Anytime), and before or after reaching 2L for patients diagnosed and died between 2013-2021.*

	Non-SCT			SCT		
**Immediate cause of death**	**Anytime n (%)**	**Death before 2L n (%)**	**Death after 2L n (%)**	**Anytime n (%)**	**Death before 2L n (%)**	**Death after 2L n (%)**
**N**	553	242	311	87	14	73
**Other baseline malignancy**	27 (5)	22 (9)	5 (2)	0 (0)	0 (0)	0 (0)
**CVD**	50 (9)	26 (11)	24 (8)	6 (7)	≤5	≤5
**Infection**	100 (18)	56 (23)	44 (14)	18 (21)	6 (43)	12 (16)
**MM**	304 (55)	108 (45)	196 (63)	57 (66)	4 (29)	53 (73)
**Other**	45 (8)	17 (7)	28 (9)	<5	≤3	≤3
**Secondary malignancy**	27 (5)	13 (5)	14 (5)	<3	<3	0 (0)

Supplementary Table 6*. Age and causes of death for those non-SCT patients who got diagnosed and died in 2013-2021 and stratified by timing of the death: within 6 months of diagnosis, or later.*

		Stratification by timing of death since MM diagnosis	
**Variable**	**Estimate/level**	**Within 6 months**	**After 6 months**
n		111	442
Age in diagnosis, years	mean (SD)	76 (8)	75 (9)
	median (IQR)	76 (72, 81)	76 (70, 81)
Immedicate death cause, n (%)	Baseline malignancy	<5	24 (5)
	CVD	8 (7)	42 (10)
	Infection	26 (23)	74 (17)
	MM	59 (53)	245 (55)
	Other	13 (12)	32 (7)
	Secondary malignancy	<3	25 (6)

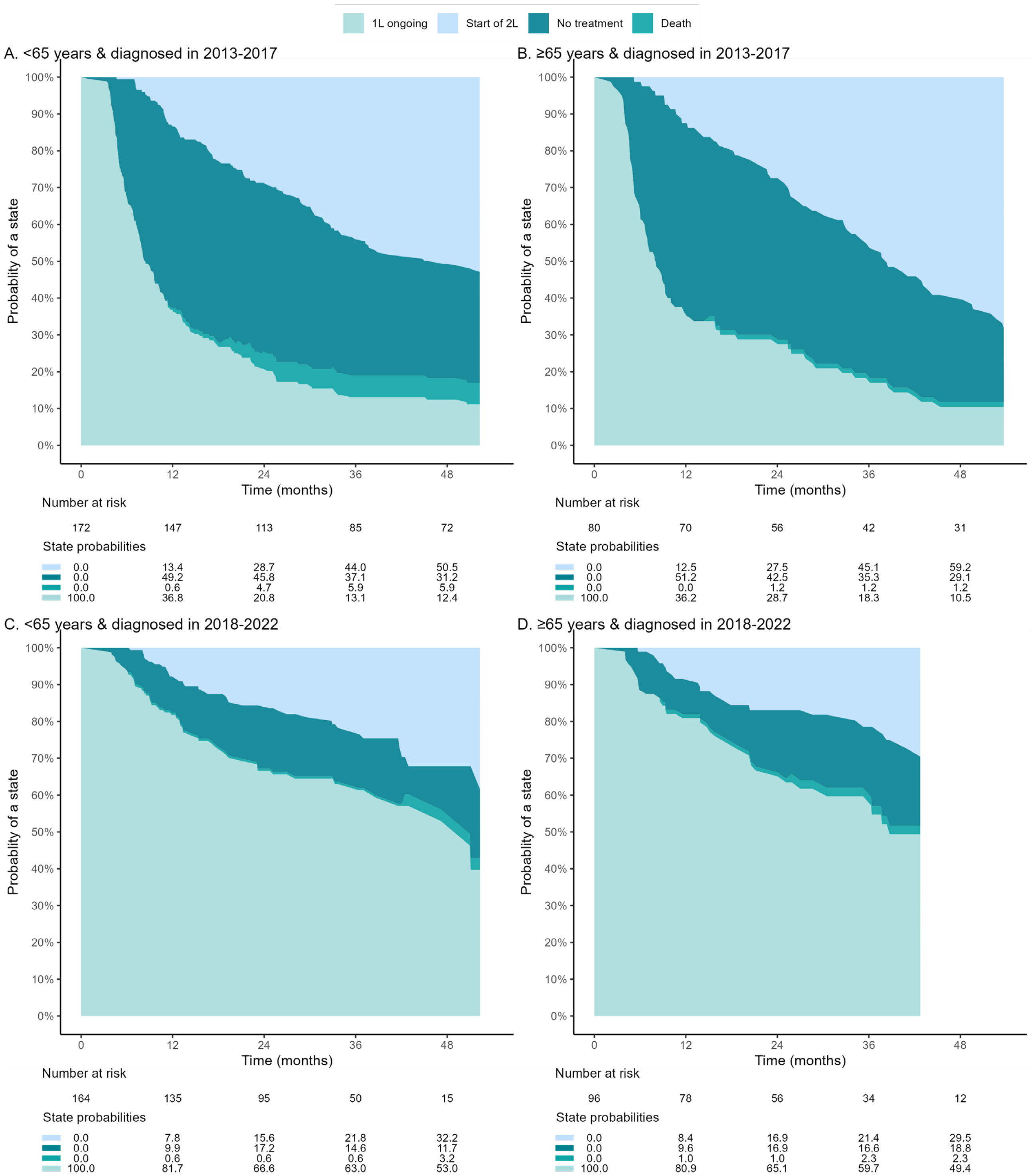

Supplementary Figure 4. *Multistate model of SCT after starting 1L treatment for patients <65 years at index (left panels) and ≥65 years at index (right panels) diagnosed between 2013-2017 (upper) and between 2018-2022 (lower panels).*

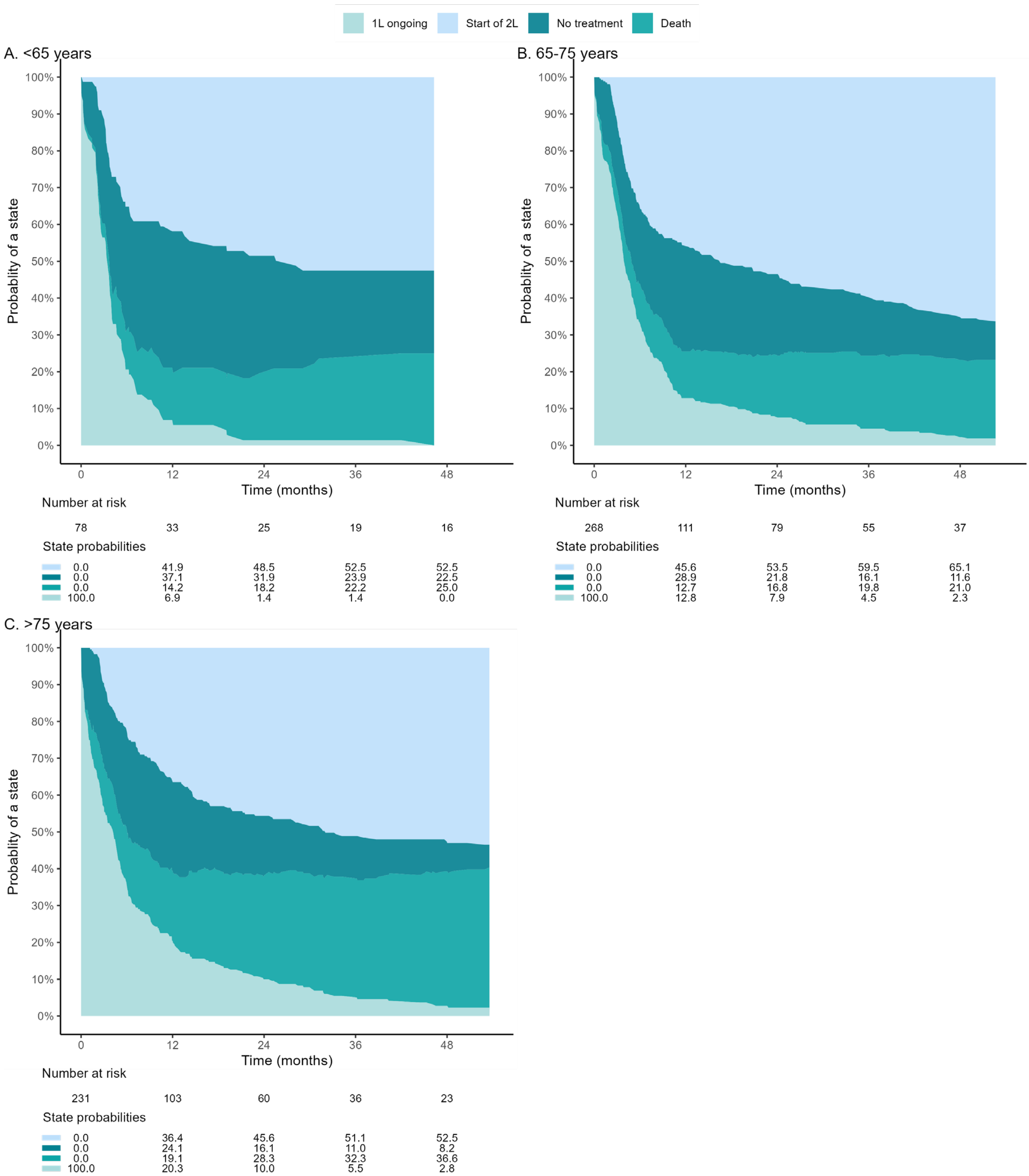

Supplementary Figure 5. *Multistate model of non-SCT patients after starting 1L treatment for patients <65 years at index (upper left panel) and 65-75 years at index (upper right panel) and ≥75 years at index for patients diagnosed between 2013-2017.*

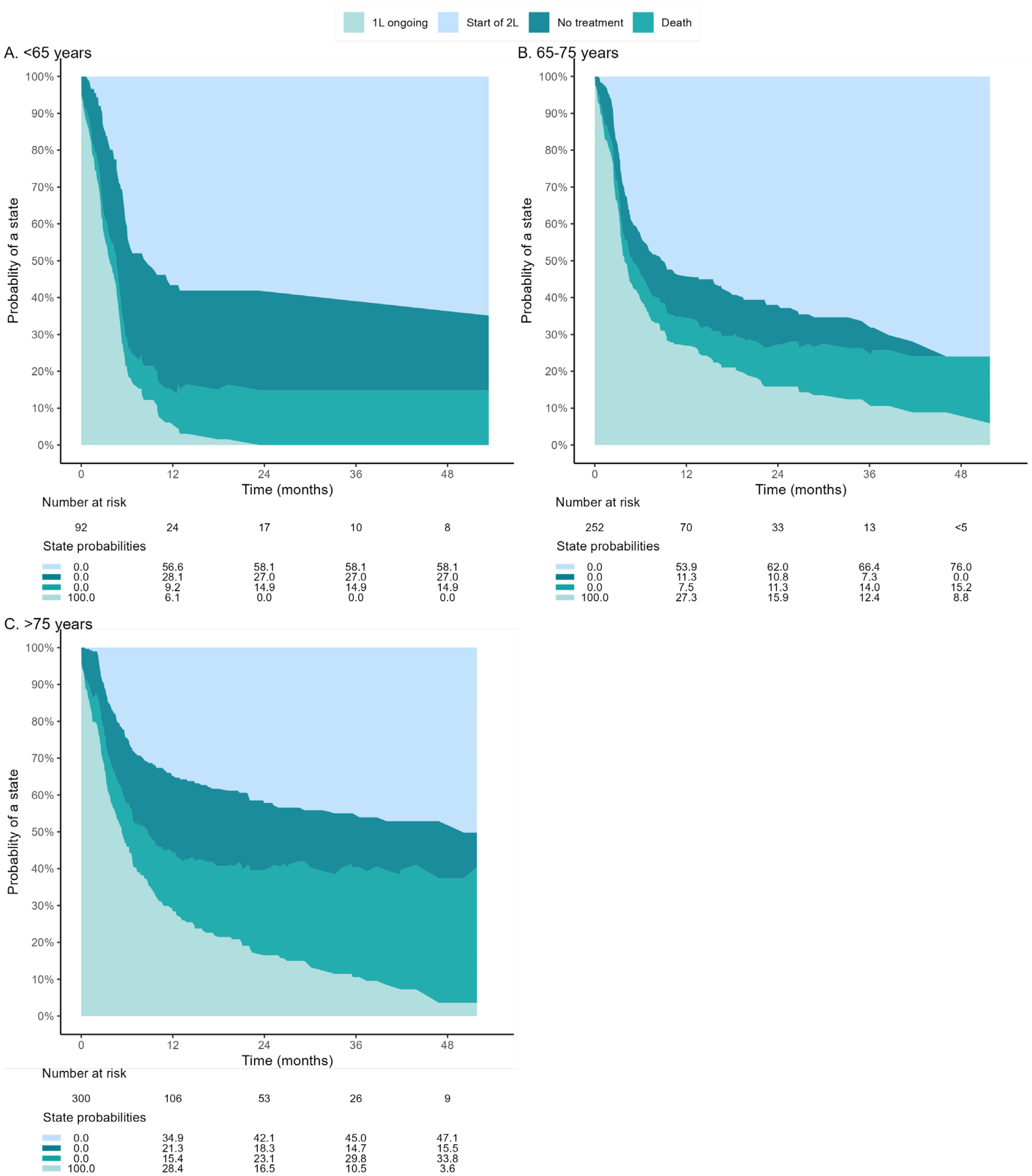

Supplementary figure 6. *Multistate model of non-SCT patients after starting 1L treatment for patients <65 years at index (upper left panel) and 65-75 years at index (upper right panel) and ≥75 years at index for patients diagnosed between 2018-2022.*

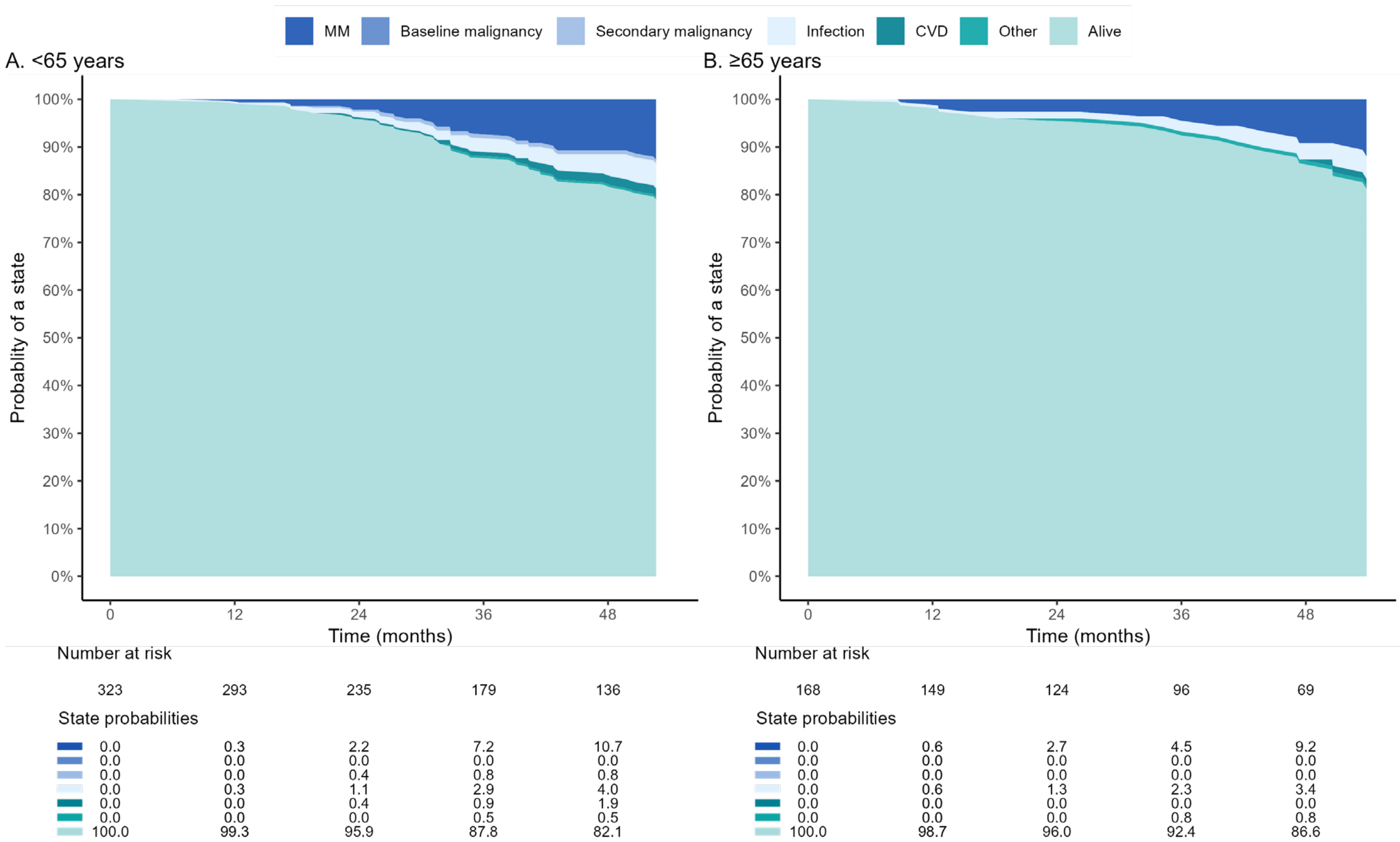

Supplementary figure 7*. Mortality and immediate causes of death for SCT patients for patients <65 years at index (left panel) and ≥65 years at index (right panel) for patients diagnosed between 2013-2021.*

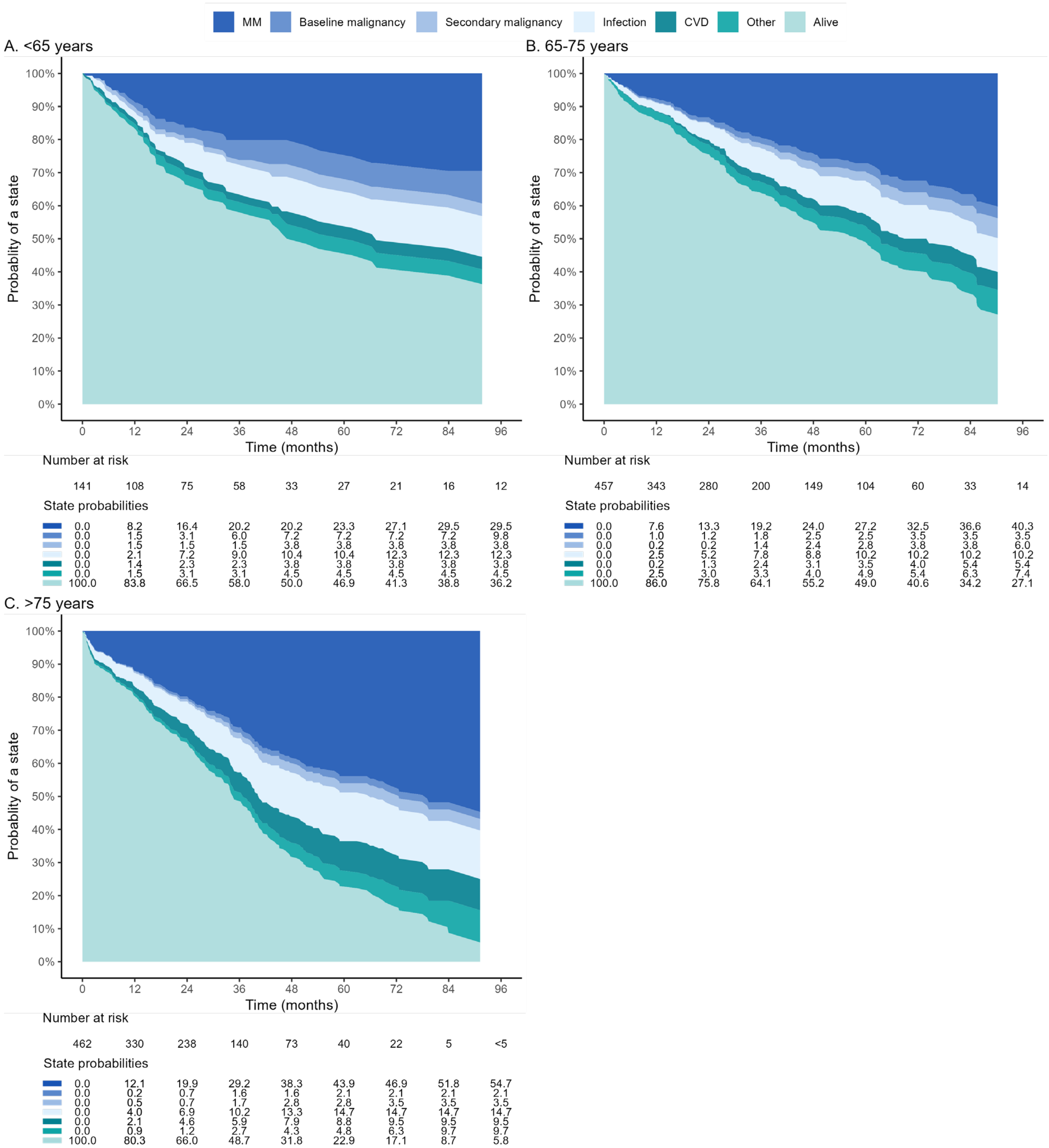

Supplementary figure 8. *Mortality and immediate causes of death for non-SCT patients for patients <65 years at index (upper left panel) and 65-75 years at index (upper right panel) and ≥75 years at index for patients diagnosed between 2013-2021.*
